# The mediating role of mentalization and integrative self-knowledge in the relationship between childhood trauma and fear of intimacy

**DOI:** 10.3389/fpsyg.2024.1384573

**Published:** 2024-06-28

**Authors:** Seyyedeh Sara Riazi, Mehdi Manouchehri

**Affiliations:** Department of Clinical Psychology, Faculty of Medicine, Islamic Azad University of Medical Sciences, Tehran, Iran

**Keywords:** fear of intimacy, childhood trauma, mentalization, integrative self-knowledge, mediating role, clinical setting

## Abstract

**Introduction:**

Since intimacy is a fundamental human need within social relationships, and recognizing that a fear of intimacy correlates with various negative consequences, it becomes crucial to examine the origins and factors that contribute to addressing this issue. This research aimed to investigate the mediating roles of mentalization and integrative self-knowledge in the link between childhood trauma and the fear of intimacy.

**Methods:**

Conducted as correlational descriptive research, our study incorporates a total sample of 303 adult women and men participants aged 20 to 50 in Tehran using the convenience sampling method. They completed the Fear of Intimacy Scale (FIS), the Childhood Trauma Questionnaire (CTQ), the Mentalization Scale (MentS), and the Integrative Self-Knowledge Scale (ISK). To analyze the research data at the descriptive level, frequency, percentage, standard deviation, and Pearson’s correlation coefficient were used, while path analysis tested our hypotheses in SPSS version 26 and AMOS version 24. Fit indices were used to check the model’s fit, and the mediation test was performed using the bootstrapping method. The fit indices revealed an excellent fit of the model with the data (*χ*2 = 1.51, *χ*2/df = 1.51, *p* = 0.219; RMSEA = 0.05; SRMR = 0.02; CFI = 0.99; NFI = 0.99; TLI = 0.99).

**Results:**

Results indicate mentalization fully mediates the childhood trauma-fear of intimacy relationship (*β* = 0.14, *p* < 0.01). However, the indirect relationship between childhood trauma and fear of intimacy through integrative self-knowledge was insignificant. The results also showed that the path coefficient from mentalization to fear of intimacy was negative and significant (*β* = −0.41, *p* < 0.001), while the path coefficient from integrative self-knowledge to fear of intimacy was not significant (*β* = −0.02, *p* > 0.05).

**Discussion:**

Based on the current findings indicating the complete mediation of mentalization and the insignificance of the mediation of integrative self-knowledge, we can deduce that enhancing the capacity for mentalization holds promise in effectively addressing intimacy-related issues. Overall, the study suggests mentalization effectively predicts the relationship between childhood trauma and fear of intimacy. This, in turn, may mitigate the detrimental effects of challenging childhood experiences on an individual’s ability to engage in intimacy and cultivate emotional closeness.

## 1 Introduction

Presently, there is no denying that the human requirement for intimacy and attachment stands as a fundamental and primary need, and neglecting it can have detrimental psychological consequences (Baumeister and Leary, 1995). The capacity to develop close relationships with others is a crucial factor contributing to adults’ mental health and overall well-being (Hook et al., 2003). Moreover, intimacy plays a vital role in establishing productive and effective relationships. Ryff (1989) posited that a high-quality relationship is characterized by the parties’ ability to engage in interactive and reciprocal experiences of empathy, love, intimacy, and understanding (Fung, 2011).

Various definitions of intimacy exist, with Descutner and Thelen (1991) outlining three essential components that they contend must coexist for the concept of intimacy to be applicable. These components encompass *content* (the sharing of personal information), *emotional valence* (having strong feelings about sharing personal information), and *vulnerability* (holding a high regard for the other person). Similarly, Ghorbani (2018b) has proposed a similar definition of intimacy, framing it as trust that facilitates understanding and being understood. This trust creates a conducive environment for individuals to share their emotions and personal thoughts. Consequently, intimacy provides a platform for emotional closeness, allowing individuals to articulate their deepest thoughts and feelings. However, there are instances where individuals may lack the capacity for emotional intimacy, often rooted in adverse childhood experiences. The anxiety stemming from sharing personal thoughts and feelings with a significant other can lead to the repression of this capacity, resulting in an avoidance of intimacy. Termed the *fear of intimacy* by Descutner and Thelen (1991), this concept encompasses individuals who harbor apprehensions about becoming emotionally close to important ones despite their desire for intimate relationships. This fear is not confined to specific relationships and can manifest in connections with romantic partners, friends, colleagues, and others. Individuals experiencing this fear encounter challenges in sharing their personal thoughts and feelings, particularly with those they hold in high regard (Senese et al., 2020).

The fear of intimacy can be comprehended through the lens of dynamic psychotherapy, particularly in the context of childhood trauma. In the early stages of life, individuals may encounter frustration in attempting to fulfill their needs, realizing that immediate and unquestionable satisfaction of all needs is not always feasible. At times, this frustration exceeds the child’s mental capacity, resulting from numerous overwhelming psychological, physical, and sexual injuries emanating from attachment figures, typically parents (Neborsky and Ten, 2018). Confronted with these complex, conflictual, and frequent emotions, the child may find it challenging to tolerate them. Consequently, intense emotions give rise to anxiety, leading the individual to employ various destructive defense mechanisms to cope with both the anxiety and the emotions that initially triggered it. This defensive process ultimately instills a fear of establishing emotional closeness with others (Davanloo, 2001). Hence, *childhood trauma* encompasses all forms of child abuse, including neglect, physical abuse, sexual abuse, emotional abuse, and domestic violence (Bücker et al., 2012).

Aligned with the experience of childhood trauma, individuals may lose the ability to perceive their inner and outer worlds, opting to hide and repress emotions rather than engage in processing and reflection (Bateman et al., 2023). This mental process, termed *mentalization*, was initially proposed by Fonagy and Target (1997) within the framework of attachment theory. Mentalization denotes a person’s capacity to comprehend and envision the intentions and reasons underlying behaviors and feelings, both their own and those of others, fostering emotional regulation (Bateman et al., 2023). Furthermore, the ability to verbalize one’s feelings and intentions toward others constitutes a pivotal component of mentalization (Ensink et al., 2017).

Conversely, within the framework of self-knowledge, the cognizance of inner states is elucidated through the concept of *integrative self-knowledge*. Ghorbani (2018a) outlines the components of integrative self-knowledge, including attention, awareness, tolerance for processing ongoing experiences, reflection on current experiences with connections to past experiences, awakening blocked past experiences, and attributing meaning to them from a new perspective. This process involves creating a coherent life narrative and formulating intentions and motivations in line with individual needs and values. Integrative self-knowledge is bifurcated into experiential and reflective self-knowledge (Ghorbani et al., 2008). “*Experiential self-knowledge*” involves processing current experiences as they occur. In contrast, *“reflective self-knowledge*” engages in active information processing about oneself in past experiences, analyzing one’s own experiences through higher cognitive functions to provide a broader perspective for guiding behavior (Ghorbani, 2018a, p.158). These two components are considered as present- and past-oriented self-experience (Ghorbani et al., 2008). As these past and present experiences become integrated, the individual can form a coherent narrative of their life and envision a desired and stable future for themselves. This leads to a cohesive sense of self and, consequently, the experience of mental well-being (Ghorbani, 2018a).

The evidence substantiates the connection between research variables, indicating that individuals who have undergone various childhood traumas tend to avoid intimate relationships in adulthood. This relationship is underscored by studies demonstrating that childhood trauma correlates with an adult’s fear of intimacy, leading to adverse psychological consequences such as loneliness, depression, anxiety, and negative self-evaluation (Riggs, 2014; Ji et al., 2015; Rohner et al., 2019; Senese et al., 2020). Furthermore, individuals with fear of intimacy often exhibit low self-esteem, emotional isolation, and dissatisfaction in their communication with significant others (Descutner and Thelen, 1991; Doi and Thelen, 1993).

**Figure 1:**
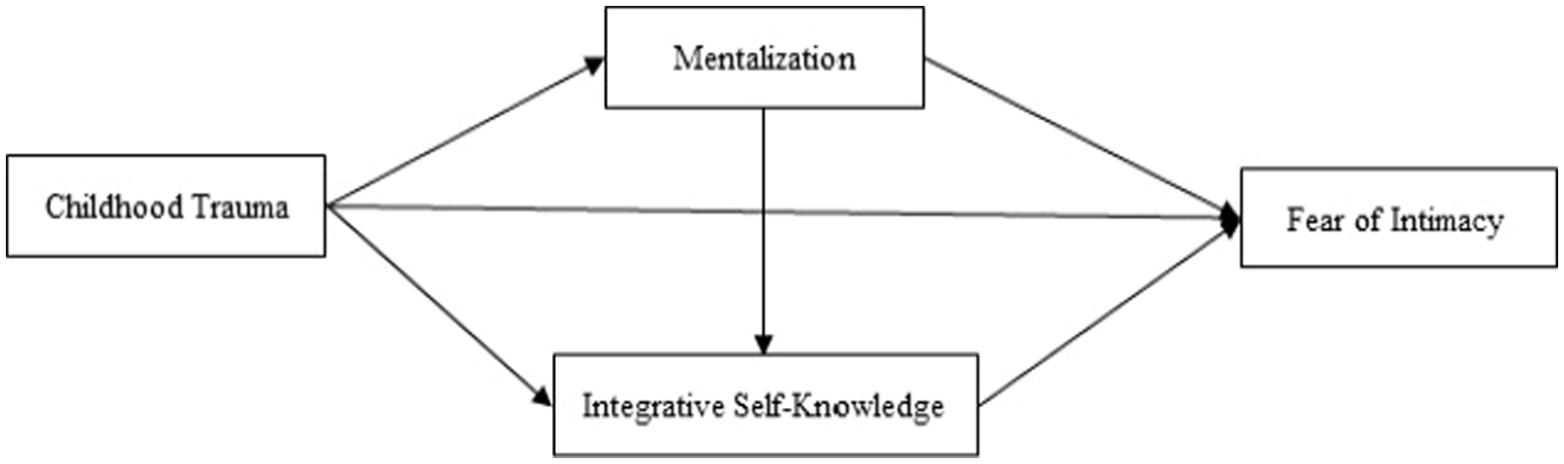
The proposed moderated mediation model.

In addition, based on research literature, one of the most pivotal mental processes associated with trauma is mentalization (Fonagy et al., 2007; cited by Ensink et al., 2017). Challenges in mentalizing have been identified as a key process through which trauma experiences can be linked to an elevated risk of developing psychopathology (Taubner and Curth, 2013; Wais, 2022). Conversely, psychological trauma impedes attachment relationships and constrains the capacity for mentalization Fonagy et al. (2018); cited in Wais (2022). Besides, numerous studies highlight the mediating role of mentalization in mitigating the detrimental impacts of childhood trauma. Mentalization is considered a critical component that, over time, can effectively contribute to self-reconstruction and enhanced emotional capacity in individuals who have experienced early adversities (Macintosh, 2013; Taubner and Curth, 2013; Ensink et al., 2017; Wais, 2022). Despite the disruption of healthy relationship patterns established during childhood, the acquired mentalizing ability can play a pivotal role in facilitating therapeutic interventions and enhancing emotional capacity, subsequently reducing the fear of intimacy (Lorenzini et al., 2018). Research indicates that fear of intimacy correlates with impaired interpersonal connections and diminished relationship satisfaction (Descutner and Thelen, 1991; Janong, 2001). Consequently, enhancing mentalizing capacity can potentially alleviate the fear of intimacy by fostering better interpersonal functioning. Additionally, Fonagy et al. (2018) underscore the interconnectedness of emotion regulation and attachment with mentalizing capacity. Given that individuals grappling with fear of intimacy often struggle with emotion regulation (Janong, 2001), it follows that enhancing mentalizing capacity could play a pivotal role in mitigating fear of intimacy through improved emotion regulation skills (Figure 1).

**Figure 2:**
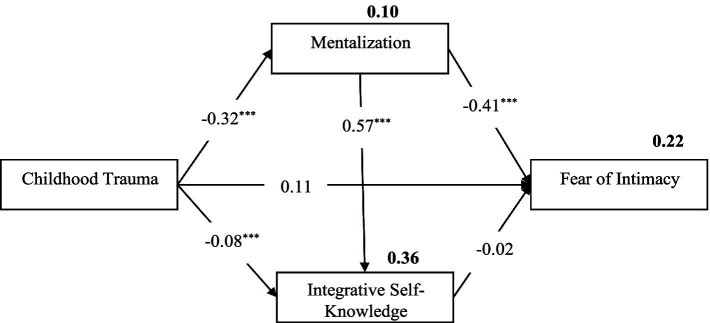
Model with standardized estimates, and proportion of explained variance. ^***^p < 0.001; proportions of explained variance are depicted in boldface.

Moreover, our childhood experiences and their impact on our development play a crucial role in shaping our self-image throughout our lives. Indeed, these early encounters serve as a fertile ground for cultivating self-awareness (St Clair, 2004). Viewed from this vantage point, our sense of identity and coherence evolves primarily through our interactions with primary caregivers (Ahrens et al., 2012). When a child’s self-perception is shattered by traumatic experiences during childhood, they often internalize feelings of worthlessness, low self-esteem, unlovability, and inadequacy (Zhong, 2023). This barrage of negative emotions, directed toward both themselves and their parents, leads to a disruption of their sense of coherence (Kohut, 2013). Consequently, a diminished capacity for self-awareness over time is likely to foster fear of intimacy, leading individuals to resort to harmful psychological defense mechanisms. Conversely, research indicates that heightened self-insight within interpersonal dynamics corresponds to a diminished fear of intimacy, particularly in individuals who have experienced childhood trauma (Davis et al., 2001). On the other hand, previous research has shown that fear of intimacy has significantly adverse effects on mental health, such as increased anxiety, depression, and reduced self-esteem (Descutner and Thelen, 1991). In contrast, integrative self-knowledge has been recognized as highly efficacious in enhancing overall mental well-being and effectively reducing anxiety and depression in various studies (Bijari et al., 2016; Ghorbani, 2018a). Therefore, enhanced Integrative self-knowledge capacity can contribute to alleviating the symptoms associated with fear of intimacy. Throughout life, the most intimate relationship is with oneself; however, individuals may, at times, experience alienation from themselves, resorting to mechanisms that work against their own well-being (Ghorbani, 2018b). Therefore, the fear of intimacy is not solely apprehension about establishing close connections with others; it also arises from existential fears (Firestone and Firestone, 2004). Despite these implications, there remains a notable research gap regarding integrative self-knowledge, its influence on fear of intimacy, and its role as a mediator in the context of childhood trauma (Figure 2).

The mediating variables in this study, mentalization, and integrative self-knowledge are also closely interconnected. Mentalization intersects with self-reflective capacity, mindfulness, and integrity (Luyten and Fonagy, 2015), forming the fundamental pillars of the integrative self-knowledge process. Furthermore, mentalization serves as the cornerstone for individual identity and self-awareness, nurturing a sense of agency, coherence, and continuity (Allen et al., 2003). This aligns precisely with the dual aspects of integrative self-knowledge, which involve an ongoing awareness of internal processes, assimilation of past experiences, and attribution of new perspectives to current experiences. This process fosters a sense of autonomy and agency within individuals (Ghorbani, 2018a). Indeed, the concepts of mentalization and integrative self-knowledge share significant commonalities, as both are essential for the development of a coherent sense of self.

The fear of intimacy is known to have numerous detrimental effects on the mental health of adults. However, research in this particular domain remains limited. Importantly, the roots of the fear of intimacy can be traced back to early traumatic experiences (Repic, 2007), with individuals affected by it often exhibiting resistance to change. Despite the implementation of experiential treatments, studies suggest minimal effectiveness in addressing this issue (Manbeck et al., 2020; Nabizadeh et al., 2020). Those grappling with fear of intimacy tend to rely on various immature defense mechanisms to alleviate the strain of their overwhelming experiences, which have become syntonic to their core self. As a result, the change process for these individuals unfolds gradually, necessitating an in-depth investigation considering many factors. Notably, a significant research gap exists in the exploration of influential or protective factors that may act as mediators between traumatic childhood experiences and the subsequent fear of intimacy.

This study delves into the intricate relationships between childhood trauma, fear of intimacy, mentalization, and integrative self-knowledge. While existing literature underscores the detrimental impact of childhood trauma on adult relationships and mental health, a notable research gap persists in understanding the mediating mechanisms, particularly the roles of mentalization and integrative self-knowledge. By exploring these dynamics, this research aims to shed light on the nuanced pathways linking early traumatic experiences to adult fear of intimacy, paving the way for a deeper comprehension of the underlying processes and potential interventions to address this complex issue. Therefore, the primary objective of this research is to formulate a model elucidating the mediating roles of mentalization and integrative self-knowledge in the relationship between childhood trauma and the fear of intimacy.

## 2 Methods

### 2.1 Participants and procedure

This study employed a descriptive research design, utilizing a quantitative, cross-sectional approach with correlational and path analysis techniques. The statistical population comprised all men and women aged 20 to 50 years residing in Tehran, Iran, during the year 2022. Using a convenience sampling method, 303 individuals were selected as the study sample. The appropriate sample size for path analysis was determined based on the number of observable variables within the model. Various guidelines suggest 5, 10, or 20 participants per observable variable (Kline, 2014, 2016). Given the criterion of 20 participants per observable variable and the presence of 13 observable variables, an initial sample size of 260 was calculated. However, to align with the recommendations of researchers who advocate for a minimum sample size of 300 (Tabachnick et al., 2013), the sample size was increased to 300. Anticipating potential issues with incomplete or distorted questionnaires, the sample size was further augmented to 350. Following data collection, 47 questionnaires were excluded due to incompleteness or distortion, resulting in a final sample size of 303 individuals. Of these, 206 (68%) were women, and 97 (32%) were men. The age distribution included 172 individuals (56.77%) aged 20–30, 77 individuals (25.41%) aged 31–40, and 54 individuals (17.82%) aged 41–50.

#### 2.1.1 Childhood trauma questionnaire (CTQ)

This questionnaire was developed by Bernstein et al. (2003) to assess childhood trauma and abuse. The questionnaire consists of 28 items, with 25 measuring the core components of childhood trauma and 3 designed to identify individuals who may deny their childhood problems. Before scoring, the responses to items 5, 7, 13, 19, 28, 2, and 26 must be reversed. Items 10, 16, and 22 assess the validity of responses; if the sum of these items exceeds 12, the individual’s responses are likely invalid. The CTQ is suitable for both adults and adolescents. Participants rate the frequency of childhood maltreatment by parents on a 5-point Likert scale, ranging from 1 (never) to 5 (very often). The questionnaire evaluates five types of childhood maltreatment: sexual abuse, physical abuse, emotional abuse, emotional neglect, and physical neglect. Each subscale score ranges from 5 to 25, with the total score ranging from 25 to 125. Higher scores indicate greater trauma or abuse, while lower scores suggest less childhood trauma or abuse. In Bernstein et al.’s study, Cronbach’s alpha values for the questionnaire were 0.87 for emotional abuse, 0.86 for physical abuse, 0.95 for sexual abuse, 0.89 for emotional neglect, and 0.78 for physical neglect. Concurrent validity was demonstrated, with correlations between the CTQ and clinician ratings of childhood trauma ranging from 0.59 to 0.78 (Bernstein et al., 2003). Garrusi and Nakhaee (2009) examined the questionnaire in an Iranian adolescent sample and reported internal consistency reliability with Cronbach’s alpha values greater than 0.70 for the total score and subscales. They also reported divergent validity with the General Health Questionnaire, with a correlation coefficient greater than 0.32.

#### 2.1.2 Mentalization scale

The Mentalization Scale (MentS) was developed by Dimitrijević et al. (2017) and comprises 28 items rated on a 5-point Likert scale, ranging from 1 (completely disagree) to 5 (completely agree). The questionnaire includes three subscales: Self-Related Mentalizing (8 items), Other-Related Mentalizing (10 items), and Motivation for Mentalizing (10 items). Items 8, 9, 11, 14, 18, 19, 21, 22, 26, and 27 require reverse scoring. Scores for each subscale are calculated as follows: Self-Related Mentalizing scores are derived from items 8, 11, 14, 18, 19, 21, 22, and 26; Other-Related Mentalizing scores from items 2, 3, 5, 6, 10, 12, 20, 23, 25, and 28; and Motivation for Mentalizing scores from items 1, 4, 7, 9, 13, 15, 16, 17, 24, and 27. The total score is the sum of the scores of the three subscales. The Cronbach’s alpha for the subscales and the total score ranges from 0.74 to 0.79. Dimitrijević et al. (2017) validated the questionnaire by correlating mentalizing scores with emotional intelligence and empathy variables using the NEO Five-Factor Inventory (NEO-FFI). They found that mentalizing is related to cognitive structures and the NEO-FFI, with significant positive correlations with empathy and emotional intelligence. Additionally, the scale showed negative correlations with avoidant and anxious attachment and neuroticism. Safari Mousavi et al. (2021) further examined the psychometric properties of the scale in the Iranian population in two non-clinical groups and one group with borderline personality disorder. The Cronbach’s alpha was 0.84 and 0.75 for the non-clinical groups, respectively, with subscale reliability ranging from 0.76 to 0.77. In the group with borderline personality disorder, Cronbach’s alpha was 0.79 for the Self-Related Mentalizing subscale, 0.76 for the Other-Related Mentalizing subscale, and 0.60 for the Motivation for Mentalizing subscale.

#### 2.1.3 Integrative self-knowledge scale (ISK)

This questionnaire was developed and validated by Ghorbani et al. (2008). The ISK consists of 12 closed-ended items rated on a 5-point Likert scale. Items 1, 2, 4, 5, 7, 8, 10, 11, and 12 are reverse scored. The scale measures three dimensions: reflective self-awareness, experiential self-awareness, and integrating past and present experiences to create a desired future. Specifically, items 3, 6, and 9 relate to reflective self-awareness; items 1, 5, 7, and 8 relate to experiential self-awareness; and items 2, 4, 10, 11, and 12 relate to integrating past and present experiences to create a desired future. The score range for this questionnaire is 12 to 60. Scores between 1 and 12 indicate weak integrative self-knowledge; scores between 12 and 36 indicate moderate integrative self-knowledge; and scores above 36 indicate very good integrative self-knowledge. The concurrent validity, incremental validity, and reliability (internal consistency and retest reliability) of this instrument have been confirmed in both Iranian and American samples. In a sample of 230 students from Tehran University, the reliability of the scale was demonstrated with a Cronbach’s alpha coefficient of 0.90 for experiential self-awareness and 0.84 for reflective self-awareness. The correlation between the two facets was 0.74. The retest reliability of the scale after a 7–8-week interval, with a sample of 44 participants, was 0.76 for experiential self-awareness and 0.68 for reflective self-awareness. Ghorbani et al. reported Cronbach’s alpha for the scale in three Iranian samples and three American samples as follows: 0.82 for the first Iranian sample, 0.81 for the second Iranian sample, 0.81 for the third Iranian sample, 0.78 for the first American sample, 0.78 for the second American sample, and 0.74 for the third American sample (Ghorbani et al., 2008). The study also confirmed the convergent, criterion, differential, and incremental validity of the scale. Additionally, in a study by Ghorbani et al. (2010), Cronbach’s alpha for the scale was reported to be 0.79.

#### 2.1.4 Fear of intimacy scale (FIS)

This questionnaire is a 35-item self-report measure developed by Descutner and Thelen (1991) to assess anxiety related to intimacy. Responses are rated on a 5-point Likert scale, ranging from “strongly agree” to “strongly disagree” (5 to 1 points). Items 3, 6, 7, 8, 10, 14, 17, 18, 19, 21, 22, 25, 27, 29, and 30 are reverse scored. The total possible scores range from 35 to 175, with higher scores indicating a greater fear of intimacy. To validate this scale, Descutner and Thelen employed both discriminant and convergent validity measures, using the Jourard Self-Disclosure Scale, the Miller Social Intimacy Scale, the UCLA Loneliness Scale, and the Short Form Need for Cognition (Descutner and Thelen, 1991). The Fear of Intimacy Scale showed a significant positive correlation with the UCLA Loneliness Scale (*r* = 0.48) and negative correlations with the Jourard Self-Disclosure Scale (*r* = −0.55), the Miller Social Intimacy Scale (*r* = −0.60), and the Need for Cognition Scale (*r* = −0.24), indicating favorable validity results. In terms of reliability, the initial study reported high internal consistency (Cronbach’s alpha = 0.93) and high test–retest reliability (0.89). The scale was standardized in Iran by Falahzadeh et al. (2011) on a sample of 567 individuals, comprising 329 women and 238 men. Factor analysis of the 35 items revealed two factors: Factor 1, Fear of Intimacy in “Romantic Relationships,” and Factor 2, Fear of Intimacy in “Other Relationships.” The internal consistency of the total scale was 0.83, with Factor 1 at 0.81 and Factor 2 at 0.70. The test–retest reliability coefficient for the total scale was 0.92, and for the subfactors, it was 0.87 for Factor 1 and 0.85 for Factor 2. Confirmatory factor analysis indicated that the data fit well with the proposed model (Falahzadeh et al., 2011).

### 2.2 Data analysis

The data were analyzed using SPSS 22 and Amos 22 software packages. Since answering all items was mandatory, it was unnecessary to impute missing values. The normality of the data was checked, and outliers were removed. Descriptive analysis and bivariate correlation (Pearson’s) were performed to check the hypothesized relationships. A confirmatory factor analysis (CFI) was conducted to evaluate the psychometric properties and the measurement model of the instruments used in the study for each of the constructs of childhood trauma, mentalization, integrative self-knowledge, and fear of intimacy. Before testing the path analysis model, the assumption of the absence of multicollinearity in the predictor variables was checked using two indices of variance inflation factor (VIF) and tolerance, which indicated the absence of multicollinearity. The path analysis was performed using the maximum likelihood estimation (ML) method. To evaluate the fit of the confirmatory factor analysis and path analysis model, fit indices such as CFI, TLI, NFI, SRMR, and RMSEA were used. Acceptable fit is indicated by CFI, TLI, NFI greater than 0.9, and SRMR and RMSEA smaller than 0.08 (Schreiber et al., 2006; Hair, 2014). Finally, the mediation test was performed using the bootstrapping method.

#### 2.2.1 Confirmatory factor analyses of the instruments used

The present study used four instruments to measure the four constructs of the proposed model and conducted a confirmatory factor analysis for each construct (see Table 1). The Childhood Trauma Questionnaire (CTQ) consisted of 28 items and five factors, but items 1, 6, 12, 19, and 25 were removed because they showed low factor loadings. The Integrative Self-Knowledge Scale (ISKS), consisting of 12 items assessing three factors, with all items’ factor loading >0.4. The Mentalization Questionnaire (MQ) consisted of 28 items and three factors, but four were eliminated because of low factor loadings (numbered 2, 9, 17, and 27). Furthermore, the Fear of Intimacy Scale (FIS) consists of 35 items in two factors, but items 1, 9, 14, 16, 17, 24, and 26 were rejected due to low factor loading scores.

**Table 1:** CFA’s fit indices for the instruments used.

Instruments	CFI	TLI	NFI	SRMR	RMSEA
Childhood trauma	0.92	0.90	0.90	0.05	0.08
Mentalization	0.90	0.90	0.90	0.07	0.06
Integrative self-knowledge	0.94	0.91	0.90	0.05	0.08
Fear of intimacy	0.91	0.90	0.90	0.07	0.06

A path analysis was conducted to evaluate the fit of the proposed model with the data. The fit indices revealed an excellent fit of the model with the data (χ^2^ = 1.51, χ^2^/df = 1.51, *p* = 0.219; RMSEA = 0.05; SRMR = 0.02; CFI = 0.99; NFI = 0.99; TLI = 0.99) and was able to explain 22% of the variance in the fear of intimacy.

### 2.3 Ethical declaration

During the research, all participants were fully informed about the study’s purpose, procedures, and possible risks. We also provided their voluntary consent to participate. The data collected was kept confidential and anonymized to protect participants’ privacy. The study design, data collection, and analysis procedures were conducted with transparency and objectivity, ensuring that the results accurately reflected the research question. Data were collected and analyzed with meticulous care and precision to ensure the reliability and validity of the findings.

## 3 Results

### 3.1 Preliminary results

After checking the normality of the data distribution in the sample using Mahalanobis distance, multivariate outlier data were removed. The skewness of the variables ranged from −0.38 to 1.41, and the kurtosis was between −0.91 and 1.76, indicating univariate normality (Table 2).

**Table 2:** Descriptive statistics and correlational indices among constructs.

	Mean	SD	Cronbach *α*	Skewness	Kurtosis	CT	Men	ISK	FOI
CT	1.61	0.58	0.88	1.41	1.76	1			
Men	2.28	0.47	0.82	−0.38	0.02	−0.32^**^	1		
ISK	3.44	0.72	0.84	0.09	−0.64	−0.20^**^	0.60^**^	1	
FOI	2.15	0.63	0.91	0.20	−0.91	0.24^**^	−0.045^**^	−0.29^**^	1

^**^p < 0.01 (two-tailed). CT, childhood trauma; Men, mentalization; ISK, integrative self-knowledge; FOI, fear of intimacy.

### 3.2 Descriptive statistics and bivariate correlations

Table 2 presents descriptive statistics and correlations among variables in the proposed research model. The Cronbach’s alpha coefficients for all variables range from 0.82 to 0.91, indicating a robust level of internal consistency according to the recommended criteria (Cronbach’s alpha >0.55) established by O’Rourke and Hatcher (2013). Notably, childhood trauma exhibits a positive and significant correlation with fear of intimacy, whereas mentalization and integrative self-knowledge indicate a negative and significant correlation with fear of intimacy. Additionally, a significant negative correlation exists between childhood trauma and integrative self-knowledge, as well as mentalization. The study found significant correlations between the variables in the proposed research model, indicating that the mediation hypothesis can be tested in this model.

The results showed that the path coefficients from childhood trauma to mentalization (*β* = −0.32, *p* < 0.001) and integrative self-knowledge (*β* = −0.08, p < 0.001) were negative and statistically significant. However, the path coefficient of childhood trauma to fear of intimacy was not significant (*β* = 0.11, *p* > 0.05). The path coefficient from mentalization to fear of intimacy was negative and significant (*β* = −0.41, *p* < 0.001), while the path coefficient from integrative self-knowledge to fear of intimacy was not significant (*β* = −0.02, *p* > 0.05). Additionally, the path coefficient from mentalization to integrative self-knowledge was positive and significant (*β* = 0.57, *p* < 0.001).

### 3.3 The mediation analysis

To examine the mediating role of mentalization and integrative self-knowledge in the relationship between childhood trauma and fear of intimacy, we employed the bootstrapping method, generating 2000 bootstrap samples (Table 3). The indirect effect was computed for three distinct paths, with only the indirect impact of childhood trauma on fear of intimacy through mentalization reaching statistical significance. Given that the direct path from childhood trauma to fear of intimacy did not achieve significance in the tested path analysis model, our inference is that mentalization serves as a complete mediator in this relationship.

**Table 3:** Mediation test.

path	Estimate	Lower	Upper	SE	*p*
CT Men ISK	0.143	0.063	0.250	0.047	0.001
CT Men ISK FOI	0.004	−0.033	0.043	0.018	0.780
CT ISK FOI	0.000	−0.011	0.014	0.006	0.902

CT, childhood trauma; Men, mentalization; ISK, integrative self-knowledge; FOI, fear of intimacy.

## 4 Discussion

The results of the path analysis revealed that mentalization serves as a complete mediator in the link between childhood trauma and the fear of intimacy (*β* = 0.14, *p* < 0.01). This finding aligns with prior studies emphasizing the crucial role of mentalization as a partial or complete mediator, including works by Wais (2022), Ensink et al. (2017), Taubner and Curth (2013), as well as Macintosh (2013). Specifically, Taubner and Curth (2013) demonstrated that mentalization serves as a complete mediator between early abuse experiences and aggressive behavior. Their conclusion highlighted mentalization as a protective factor, reinforcing the link between early abuse experiences and aggressive behavior. Additionally, research exploring mentalization as a protective and resilient factor in the context of psychological trauma in various populations, including children (Ensink et al., 2017; Borelli et al., 2018), adolescents (Venta et al., 2017), and adults (Berthelot et al., 2015; Ensink et al., 2015, 2023), supports these findings (Wais, 2022). Overall, mentalization is regarded as a crucial protective factor against diverse physical and psychological conditions in the context of trauma.

To elaborate on this finding, we can assert that one of the most pivotal mental processes associated with trauma is mentalization Fonagy et al. (2007); cited by Ensink et al. (2017). Challenges in mentalizing have been identified as a key process through which trauma experiences can be linked to an elevated risk of developing psychopathology (Taubner and Curth, 2013; Wais, 2022). Conversely, psychological trauma impedes attachment relationships and constrains the capacity for mentalization Ensink et al. (2017); cited in Wais (2022).

Mentalization comes into play when a maternal figure senses an emotion in the child and reflects it back to them. Fundamentally, the infant acquires an understanding of their emotional state through interactions with the mother. The caregiver’s presence, usually the mother, is crucial for the child’s mental growth. Fonagy et al. (2018) details how parents mirror a broad spectrum of emotions, ranging from tender affection to anger (Fonagy et al., 2018). In this context, if the emotions experienced during childhood are met with punishment and other negative reactions rather than being acknowledged, mirrored, and understood by the parents, the child may perceive closeness as perilous. Consequently, in adulthood, when attempting to establish intimacy, they may encounter anxiety and communication difficulties, reigniting past traumas (Abbass, 2015).

From a clinical perspective, it becomes crucial to identify the processes involved in post-traumatic recovery. Intense and overwhelming emotions, exceeding one’s capacity may coincide with physical harm (Frederickson, 2021). Furthermore, it is often necessary to grieve a former self without experiencing trauma. This involves accessing current experiences through the lens of past evolutionary processes, which require understanding and verbalizing emotions and mental states within a non-judgmental, secure, open, and accepting therapeutic relationship. This process is what fosters hope for future intimacy in traumatized patients (Wise, 2016).

Moreover, we hypothesized that integrative self-knowledge, as a mediating variable, can elucidate the relationship between childhood trauma and fear of intimacy. However, the path analysis results led to the rejection of this hypothesis (β = 0.00, *p* = 0.9), indicating that integrative self-knowledge, in a mediating role, cannot account for the connection between childhood trauma and fear of intimacy.

The scarcity of research on the mediating role of integrative self-knowledge in the context of childhood trauma and fear of intimacy is notable. Few studies have explored this aspect, making it challenging to assess compatibility with existing research. This study is one of the first to address this gap and represents a pioneering attempt to investigate this aspect. Nevertheless, this study may resonate with the conclusions drawn by Goodarzi et al. (2018), who suggested that integrative self-knowledge may not play a significant mediating role in the link between childhood trauma and borderline personality traits. Consequently, one could posit that integrative self-knowledge lacks robust mediation in its association with childhood trauma experiences.

The current study reveals that introducing the mentalization variable into the model neutralizes the mediating effect of self-knowledge in favor of mentalization. This indicates that mentalization incorporates aspects of self-knowledge and overlaps with self-reflective capacity, mindfulness, and identity integration (Luyten and Fonagy, 2015). These three components form the essential foundations of the integrative self-knowledge process (Ghorbani, 2018a). Mentalization refers to the understanding of one’s own and others’ experiences and behaviors through mental processes such as assumptions, attitudes, emotions, desires, knowledge, intentions, plans, dreams, hopes, and even false beliefs (Bateman and Fonagy, 2019). It serves as the cornerstone of individual identity and self-awareness, fostering a sense of agency, integrity, and continuity. Mentalization empowers individuals to recognize their behaviors and assume accountability for their decisions and actions (Allen et al., 2003), which is intertwined with integrative self-knowledge. Hence, mentalization lays the groundwork for subsequent self-knowledge. In fact, self-knowledge is better understood as the awareness of internal states rather than external ones. It empowers individuals to perceive differentiation, continuity, integrity, and interconnectedness within themselves (Ghorbani, 2018a).

In essence, mentalization emerges as a multifaceted construct, bridging both the internal and external dimensions, involving not only self-awareness but also the understanding of others (Bateman et al., 2023). Research indicates that childhood trauma also exerts a multifaceted influence on individuals, impacting both their internal and external worlds as well as their interpersonal relationships (Ensink et al., 2017). This suggests that childhood trauma may be more strongly associated with symptoms of fear of intimacy through its negative impact on children’s mentalization. As well, previous studies have underscored the significance of mentalization in the context of trauma (Taubner and Curth, 2013; Ensink et al., 2017; Wais, 2022), yet there remains a gap in research concerning the role of integrative self-knowledge in trauma contexts and its implications for interpersonal relationships and intimacy. The current finding underscores the paramount importance of the mentalization component, emphasizing the critical need for attention to this structure in both clinical and research settings. Future researchers should explore the mediating role of integrative self-knowledge in different models and within the trauma context to address the existing research gap.

Furthermore, our study’s findings demonstrated the non-significance of the direct effect of childhood trauma on fear of intimacy. This implies that, according to our research, childhood trauma is only indirectly associated with fear of intimacy through mentalization. This finding is consistent with various studies indicating an indirect rather than a direct relationship between childhood trauma or related factors, such as recalled childhood parental rejection and childhood maltreatment and fear of intimacy or intimacy perception (Williamson, 2009; Ji et al., 2015; Senese et al., 2020). It is also in line with research emphasizing the pivotal role of mentalization as a mediating factor in mitigating or eliminating the adverse effects of childhood challenges (Macintosh, 2013; Taubner and Curth, 2013; Ensink et al., 2017).

For instance, Senese et al. (2020) found that adults’ memories of parental acceptance and rejection in childhood are only indirectly linked to fear of intimacy in adulthood through psychological maladjustment after accounting for gender and age. Similarly, Ji et al. (2015) demonstrated in their study that childhood trauma and fear of intimacy are interconnected solely through feelings of inferiority, and the direct relationship between these two variables is insignificant. Taubner and Curth (2013) also concluded that mentalization serves as a complete mediator between childhood trauma and interpersonal problems in adulthood, establishing the link between childhood trauma and later aggressive behavior only indirectly.

The deepest sense of intimacy is often encountered within a mother’s embrace. However, when this sanctuary turns into a source of pain and threat, the initial emotional wound becomes a major impediment to adult intimacy (Abbass, 2015). Unconsciously, individuals tend to reenact their childhood traumas, setting the stage for mutual disappointment. Defense mechanisms developed during these early experiences later contribute to a pervasive fear of intimacy across various contexts (Repic, 2007). For many, adverse childhood experiences and subsequent failed adult relationships reinforce the belief that intimacy is either illusory, unreal or inevitably laden with pain and disappointment (Vangelisti and Beck, 2007). Childhood traumatic and stressful experiences cast a long shadow on human life, resulting in increased anxiety, impaired emotional regulation, depression, substance abuse, compulsive reliving of traumatic scenarios, and feelings of shame and guilt (Davis et al., 2001; Repic, 2007; Williamson, 2009; Miller-Perrin and Perrin, 2013; Abdul et al., 2018; Van Meter et al., 2020).

Conversely, evidence suggests that the activation of the attachment system correlates with the deactivation of mentalization. Neuroimaging studies (Nolte et al., 2013) indicate that brain regions linked to romantic and maternal attachment suppress activity in areas associated with cognitive control, including those related to mentalization and social judgments (Vrtička and Vuilleumier, 2012). Anything triggering the attachment system, along with stress-induced arousal, tends to diminish mentalizing performance. Attachment trauma can lead to chronic activation of this system. Hyperactivity of the attachment system in those who have experienced trauma may elucidate the diminished ability to mentalize in emotional situations (Luyten and Fonagy, 2015). Attachment trauma induces hyperactivity of the attachment system as the individual the child seeks in moments of anxiety (attachment figure) is the same person who initially caused fear (Fonagy et al., 2007). Failures in mentalizing during crucial moments hinder effective communication in attachment relationships (Luyten and Fonagy, 2015). Thus, experiencing attachment trauma in childhood, which compromises mentalization ability, perpetuates a persistent fear in emotional and intimate relationships. This process distinctly illustrates the sequence of relationship deterioration and the emergence of fear of intimacy due to childhood trauma and the breakdown of mentalizing ability. It underscores the pivotal role of mentalization as a complete mediator in this intricate dynamic.

Unexpectedly, our study revealed that integrative self-knowledge lost its significance in predicting fear of intimacy when mentalization was included in the model (*β* = −0.02, *p* > 0.05), despite initially showing a weak but notable correlation (*r* = −0.29, *p* < 0.01). This suggests that childhood trauma may impact fear of intimacy more through impaired mentalization than reduced self-knowledge. Previous research has consistently highlighted mentalization’s pivotal role in the context of trauma, while the role of self-knowledge in trauma and intimacy remains understudied.

Nevertheless, the initial negative correlation between self-knowledge and fear of intimacy, although lacking directly comparable studies, aligns with research on self-knowledge’s role in fulfilling basic psychological needs and enhancing life satisfaction (Ghorbani et al., 2010; Behjati et al., 2011; Ghorbani, 2018a; Hajifathali et al., 2021). As intimacy is a fundamental need (Baumeister and Leary, 1995), self-knowledge likely influences the ability to meet this need. Moreover, fear of intimacy is linked to adverse mental health effects like anxiety and depression (Descutner and Thelen, 1991), which self-knowledge has been shown to alleviate (Ghorbani et al., 2010; Behjati et al., 2011; Hajifathali et al., 2021). Moreover, evidence suggests that fear of intimacy is linked to adverse effects on mental health, including anxiety, depression, and low self-esteem (Descutner and Thelen, 1991). Conversely, multiple studies have affirmed the role of integrative self-knowledge in promoting mental health and alleviating anxiety and depression (Ghorbani et al., 2010; Behjati et al., 2011; Hajifathali et al., 2021). Therefore, integrative self-knowledge could be deemed effective in mitigating the symptoms associated with fear of intimacy.

Furthermore, object relations theory highlights how self-knowledge is constructed through interactions (Mitchell, 1991; Klein, 2010). The self is viewed as multiple and discontinuous (Mitchell, 1991). Our self-perception becomes discontinuous as our personality and identity form through engagements with diverse individuals, manifesting in different self-experiences with each person (Klein, 2010). Self-knowledge is derived from these interactions, leading to a stable identity core yet yielding diverse self-organizations centered around representations of both self and others.

Mitchell (1991) argues that our self-views are rooted in past relationships, as our connection with others shapes who we are. Viewing the self as existing across time, rather than a fixed point in space, implies it has multiple functions. Authenticity, expressed through spontaneously revealing genuine thoughts and emotions aligned with our interpersonal and internal emotional context (St Clair, 2004), underscores how past experiences of intimacy influence how we understand ourselves.

Reciprocity is key, as attributing distorted meaning to relationships, particularly with attachment figures, can disrupt self-perception and interpersonal relating. The more incoherent and fragmented one’s identity, the greater the relational confusion. Intimacy may then trigger memories of past painful relational experiences and an insecure, worthless self-view, prompting self-protective avoidance of closeness (Kohut, 2013; Frederickson, 2013; Abbass, 2015; McWilliams, 2020). Our self-understanding is interwoven with others, justifying why the multidimensional mentalization construct encompassing self-and other-oriented facets (Fonagy et al., 2018) more closely relates to intimacy components in attachment contexts than self-knowledge alone.

## 5 Conclusion

The findings of the present study demonstrate that mentalization fully mediates the relationship between childhood trauma and fear of intimacy. This implies that the direct effect of childhood trauma on fear of intimacy is neutralized when mentalization is introduced as a variable. Additionally, integrative self-knowledge failed to mediate the relationship between childhood trauma and fear of intimacy. Furthermore, the study revealed that once mentalization and integrative self-knowledge were included in the model, the direct effect of integrative self-knowledge on fear of intimacy became insignificant. The results also indicated a direct and significant relationship between mentalization and fear of intimacy. Ultimately, based on the current findings indicating the complete mediation of mentalization and the insignificance of the mediation of integrative self-knowledge, we can deduce that enhancing the capacity for *mentalization* is a crucial step in resolving emotionally resistant problems within psychodynamic psychotherapy sessions. This involves creating conditions in the psychotherapy room that allow for the processing, understanding, and experiencing emotions in a safe and receptive environment. The therapist’s ongoing reflection of the patient’s thoughts and emotions from moment to moment becomes a pivotal element in this process, potentially addressing emotional challenges that prove resistant to conventional therapy.

## 6 Limitations and strengths

This study is subject to various limitations, which we would like to discuss in the following section. First, this study is limited by self-reported measures, which may result in common method variance. Second, the study included 303 participants. A larger sample size could contribute to greater generalizability. Third, regarding sample collection, it is essential to note that our results possess limited generalizability as we exclusively utilized samples from the normal Iranian population. Fourth, another aspect to consider regarding sample collection is the sampling method employed in the present study. The non-randomized, convenience sampling method utilized poses a challenge in generalizing the results. Fifth, the sample in the present study had an unequal number of men and women. Therefore, generalizing the effect of gender differences and predicting the contribution of gender differences to adult fear of intimacy may be problematic. Last but not least, the cross-sectional survey design of this study limits the ability to establish cause-and-effect relationships. Nevertheless, it is crucial to recognize that the choice of research design, whether cross-sectional or longitudinal, is influenced by the research objectives, questions, and available resources. For this study, a cross-sectional design was adopted due to constraints in resources and time. Although a cross-sectional approach does not allow for the assessment of causal relationships or changes in variables over time, it effectively identifies correlational relationships between variables. This preliminary insight is valuable for informing the design of future longitudinal studies.

The strengths of the study may include the following: One of the strengths of this study is its novelty. It is the first to examine the mediating roles of mentalizing and integrative self-knowledge—two important dynamic concepts—in the relationship between childhood trauma and fear of intimacy. Previous studies have not addressed the influence of these two variables on fear of intimacy. Another strength lies in the use of the Mentalization Scale (MentS) developed by Dimitrijević et al. (2017). Most previous research has relied on the Reflective Functioning Scale (RFS) to assess mentalizing capacity. However, the lack of a direct and unique assessment tool for this construct posed challenges for researchers (Choi-Kain and Gunderson, 2008). The MentS not only includes more comprehensive items to assess mentalizing but also provides a more precise, cost-effective, and time-efficient tool for researchers. Finally, the findings from this study can serve as a foundation for developing educational and therapeutic programs aimed at improving intimate relationships. Additionally, these findings can contribute to the expansion of psychodynamic and experiential therapies, encouraging therapists and clinicians to incorporate more effective interventions, such as mentalization-based interventions.

## 7 Future implications

Notwithstanding the limitations of the current study, the results have several important research and practice implications. First, there is an urgent need for longitudinal research with large samples from diverse adult subpopulations to delineate the long-term consequences of childhood maltreatment and identify adults at risk of fear of intimacy and relational isolation. Such studies would not only inform practice but also provide essential evidence to support interventions aimed at reducing fear of intimacy in these subpopulations. Second, it is imperative to employ dynamic and experiential tools to accurately measure complex concepts like mentalizing. For instance, creating a clinical setting for qualitative interviews and experimentally assessing the symptoms of fear of intimacy is essential. Self-report tools alone often fall short of facilitating a thorough examination of such intricate concepts. To address this limitation comprehensively, future research should incorporate additional assessment methods. Qualitative approaches, such as third-party observation and focus group interviews, can enhance the generalizability and validity of the study’s conclusions. Finally, given the significant impact of mentalizing as a psychodynamic concept on reducing fear of intimacy, we recommend incorporating interventions aimed at strengthening mentalizing capacity into psychodynamic psychotherapy. These interventions could play a crucial role in mitigating fear of intimacy and improving relational outcomes.

## Data availability statement

The datasets presented in this article are not readily available because The data is confidential. Requests to access the datasets should be directed to manoucheri1978@gmail.com.

## Ethics statement

The studies involving humans were approved by the Department of Clinical Psychology, Azad University of Medical Sciences, Faculty of Medicine, Tehran, Iran. The studies were conducted in accordance with the local legislation and institutional requirements. The participants provided their written informed consent to participate in this study.

## Author contributions

SR: Conceptualization, Investigation, Methodology, Project administration, Resources, Writing – original draft, Writing – review & editing. MM: Conceptualization, Investigation, Methodology, Project administration, Supervision, Writing – review & editing.

## References

[ref1] Abbass A. (2015). Reaching through resistance: Advanced psychotherapy techniques. United States of America, Kansas City, Missouri: Seven Leaves Press.

[ref38] Abdul K. Sadiq H. Sana G. Samar Z. (2018). Relations between remembered childhood parental acceptance-rejection, current fear of intimacy, and psychological adjustment among pakistani adults. Psychol Behav. Sci. Int. J. 10:555784. doi: 10.19080/pbsij.2018.09.555784

[ref2] Ahrens K. R. Ciechanowski P. Katon W. (2012). Associations between adult attachment style and health risk behaviors in an adult female primary care population. J. Psychosom. Res. 72, 364–370. doi: 10.1016/j.jpsychores.2012.02.002, PMID: 22469278 PMC3816981

[ref3] Allen J. G. Bleiberg E. Haslam-Hopwood G. (2003). Mentalizing as a compass for treatment. Bull. Menn. Clin. 67, 91–112. doi: 10.1521/bumc.67.2.91.23440 14604096

[ref4] Bateman A. Campbell C. Fonagy P. Luyten P. Debbané M. (2023). Cambridge guide to Mentalization-based treatment (MBT): Cambridge University Press.

[ref5] Bateman A. Fonagy P. (Eds.) (2019). Handbook of mentalizing in mental health practice. Washington (D.C.): American Psychiatric Association Publishing.

[ref6] Baumeister R. F. Leary M. R. (1995). The need to belong: desire for interpersonal attachments as a fundamental human motivation. Psychol. Bull. 117, 497–529. doi: 10.1037/0033-2909.117.3.497, PMID: 7777651

[ref7] Behjati Z. Saeedi Z. Noorbala F. Enjedani E. Meybodi F. A. (2011). Integrative self-knowledge and mental health. Procedia Soc. Behav. Sci. 30, 705–708. doi: 10.1016/j.sbspro.2011.10.137

[ref8] Bernstein D. P. Stein J. A. Newcomb M. D. Walker E. Pogge D. Ahluvalia T.. (2003). Development and validation of a brief screening version of the childhood trauma questionnaire. Child Abuse Negl. 27, 169–190. doi: 10.1016/s0145-2134(02)00541-0, PMID: 12615092

[ref9004] Berthelot N. Ensink K. Bernazzani O. Normandin L. Luyten P. Fonagy P. (2015). Intergenerational transmission of attachment in abused and neglected mothers: The role of trauma‐specific reflective functioning. Infant Ment. Health J. 36, 200–212. doi: 10.1002/imhj.21499 25694333

[ref9] Bijari A. F. Hosseini S. H. Nasiri M. (2016). The relationship between childhood trauma, attachment style and self-knowledge in people with borderline personality disorder. J. Babol Univ. Med. Sci. 18, 14–18. doi: 10.22088/jbums.18.7.14

[ref10] Borelli J. L. Ensink K. Hong K. Sereno A. T. Drury R. Fonagy P. (2018). School-aged children with higher reflective functioning exhibit lower cardiovascular reactivity. Front. Med. 5:196. doi: 10.3389/fmed.2018.00196, PMID: 30035111 PMC6043676

[ref11] Bücker J. Kapczinski F. Post R. Ceresér K. M. Szobot C. Yatham L. N.. (2012). Cognitive impairment in school-aged children with early trauma. Compr. Psychiatry 53, 758–764. doi: 10.1016/j.comppsych.2011.12.006, PMID: 22300905

[ref12] Choi-Kain L. W. Gunderson J. G. (2008). Mentalization: ontogeny, assessment, and application in the treatment of borderline personality disorder. Am. J. Psychiatry 165, 1127–1135. doi: 10.1176/appi.ajp.2008.07081360, PMID: 18676591

[ref13] Davanloo H. (2001). Intensive short-term dynamic psychotherapy: Selected papers of Habib Davanloo. England, Chichester: Wiley.

[ref14] Davis J. L. Petretic-Jackson P. A. Ting L. (2001). Intimacy dysfunction and trauma symptomatology: long-term correlates of different types of child abuse. J. Trauma. Stress. 14, 63–79. doi: 10.1023/a:1007835531614

[ref15] Descutner C. J. Thelen M. H. (1991). Development and validation of a fear-of-intimacy scale. Psychol. Assess. 3, 218–225. doi: 10.1037/1040-3590.3.2.218

[ref16] Dimitrijević A. Hanak N. Altaras Dimitrijević A. Jolić Marjanović Z. (2017). The Mentalization scale (MentS): a self-report measure for the assessment of Mentalizing capacity. J. Pers. Assess. 100, 268–280. doi: 10.1080/00223891.2017.1310730, PMID: 28436689

[ref17] Doi S. C. Thelen M. H. (1993). The fear-of-intimacy scale: replication and extension. Psychol. Assess. 5, 377–383. doi: 10.1037/1040-3590.5.3.377

[ref9003] Ensink K. Bégin M. Martin-Gagnon G. Biberdzic M. Berthelot N. Normandin L.. (2023). Post-traumatic-stress in the context of childhood maltreatment: pathways from attachment through mentalizing during the transition to parenthood. Front. Psychol. 14:919736. doi: 10.3389/fpsyg.2023.919736 37359870 PMC10289889

[ref18] Ensink K. Bégin M. Normandin L. Godbout N. Fonagy P. (2017). Mentalization and dissociation in the context of trauma: implications for child psychopathology. J. Trauma Dissociation 18, 11–30. doi: 10.1080/15299732.2016.1172536, PMID: 27070273

[ref9002] Ensink K. Normandin L. Target M. Fonagy P. Sabourin S. Berthelot N. (2015). Mentalization in children and mothers in the context of trauma: An initial study of the validity of the Child Reflective Functioning Scale. Br. J. Dev. Psychol. 33, 203–217. doi: 10.1111/bjdp.12074 25483125

[ref19] Falahzadeh H. Farzad V. Falahzadeh M. (2011). A study of the psychometric characteristics of fear of intimacy scale (FIS). J. Res. Psychol. Health 5, 70–79.

[ref20] Firestone R. W. Firestone L. (2004). “Methods for overcoming the fear of intimacy,” in Handbook of closeness and intimacy. eds. Mashek D. J. Aron A. P. (New York, NY: Lawrence Erlbaum Associates Publishers), 375–395.

[ref21] Fonagy P. Gergely G. Jurist E. L. (2018). Affect regulation, mentalization, and the development of the self. London: Routledge.

[ref22] Fonagy P. Gergely G. Target M. (2007). The parent-infant dyad and the construction of the subjective self. J. Child Psychol. Psychiatry 48, 288–328. doi: 10.1111/j.1469-7610.2007.01727.x, PMID: 17355400

[ref23] Fonagy P. Target M. (1997). Attachment and reflective function: their role in self-organization. Dev. Psychopathol. 9, 679–700. doi: 10.1017/s0954579497001399, PMID: 9449001

[ref24] Frederickson J. (2013). Co-creating change: Effective dynamic therapy techniques. United States of America, Kansas City, MO: Seven Leaves Press.

[ref25] Frederickson J. (2021). Co-creating safety: Healing the fragile patient. United States of America, Kansas City, MO: Seven Leaves Press.

[ref26] Fung C. (2011). Exploring individual self-awareness as it relates to self-acceptance and the quality of interpersonal relationships (master’s thesis). Pepperdine University, Malibu, CA: Pepperdine Digital Commons. 195. Available at: https://digitalcommons.pepperdine.edu/etd/195

[ref27] Garrusi B. Nakhaee N. (2009). Validity and reliability of a Persian version of the childhood trauma questionnaire. Psychol. Rep. 104, 509–516. doi: 10.2466/pr0.104.2.509-516, PMID: 19610481

[ref28] Ghorbani N. (2018a). Styles and skills of interpersonal relationships. Iran: University of Tehran.

[ref29] Ghorbani N. (2018b). Self-narrated by self. Iran: University of Tehran.

[ref30] Ghorbani N. Cunningham C. J. L. Watson P. J. (2010). Comparative analysis of integrative self-knowledge, mindfulness, and private self-consciousness in predicting responses to stress in Iran. Int. J. Psychol. 45, 147–154. doi: 10.1080/00207590903473768, PMID: 22043895

[ref31] Ghorbani N. Watson P. J. Hargis M. B. (2008). Integrative self-knowledge scale: correlations and incremental validity of a cross-cultural measure developed in Iran and the United States. J. Psychol. 142, 395–412. doi: 10.3200/jrpl.142.4.395-412, PMID: 18792651

[ref32] Goodarzi N. Komijani A. H. Doniavi V. (2018). Investigating the moderating role of self-knowledge processes and self-control in the relationship between childhood traumas and the severity of borderline personality traits. Ann. Med. Health Sci. Res. 8, 257–261.

[ref33] Hair J. F. (2014). A primer on partial least squares structural equations modeling (PLS-SEM). USA, Los Angeles: Sage.

[ref34] Hajifathali F. Ghorbani N. Rostami R. (2021). The relationship between integrative self-knowledge, mindfulness, self-control, and mental health parameters. Propósitos Y Representaciones 9, 1–10. doi: 10.20511/pyr2021.v9nspe3.1277

[ref35] Hook M. K. Gerstein L. H. Detterich L. Gridley B. (2003). How close are we? Measuring intimacy and examining gender differences. J. Couns. Dev. 81, 462–472. doi: 10.1002/j.1556-6678.2003.tb00273.x

[ref36] Janong H. (2001). “The influences of parent-child affectional bonding and self-esteem on fear of intimacy in young adults (master’s thesis),” in Research Online Institutional Repository. Australia, Joondalup: Edith Cowan University. 1049. Available at: https://ro.ecu.edu.au/theses/1049

[ref37] Ji Y. Zhao B. Ma Y. (2015). Mediating effect of inferiority feelings on relationship between childhood trauma and fear of intimacy in college students. Chin. Ment. Health J. 29, 457–462.

[ref39] Klein S. B. (2010). The self: as a construct in psychology and neuropsychological evidence for its multiplicity. Wiley Interdiscip. Rev. Cogn. Sci. 1, 172–183. doi: 10.1002/wcs.25, PMID: 26271232

[ref40] Kline P. (2014). An easy guide to factor analysis. London: Routledge.

[ref41] Kline R. B. (2016). Principles and practice of structural equation modeling. New York: The Guilford Press.

[ref42] Kohut H. (2013). The analysis of the self: A systematic approach to the psychoanalytic treatment of narcissistic personality disorders. US, Chicago: University of Chicago Press.

[ref43] Lorenzini N. Campbell C. Fonagy P. (2019). “Mentalization and its role in processing trauma,” in Approaches to psychic trauma: Theory and practice. ed. Huppertz B. (Maryland, Lanham: Rowman & Littlefield), 403–422.

[ref44] Luyten P. Fonagy P. (2015). The neurobiology of mentalizing. Personal. Disord. Theory Res. Treat. 6, 366–379. doi: 10.1037/per0000117 26436580

[ref45] Macintosh H. (2013). Mentalizing and its role as a mediator in the relationship between childhood experiences and adult functioning: exploring the empirical evidence. Psihologija 46, 193–212. doi: 10.2298/psi1302193m

[ref46] Manbeck K. E. Kanter J. W. Kuczynski A. M. Maitland D. W. Corey M. (2020). Fear-of-intimacy in the interpersonal process model: an investigation in two parts. J. Soc. Pers. Relat. 37, 1317–1339. doi: 10.1177/0265407519898267

[ref47] McWilliams N. (2020). Psychoanalytic diagnosis: Understanding personality structure in the clinical process. S.L.: The Guilford Press.

[ref48] Miller-Perrin C. L. Perrin R. D. (2013). Child maltreatment: an introduction (3rd ed.). Thousand Oaks, CA: Sage Publications.

[ref49] Mitchell S. A. (1991). Contemporary perspectives on self: toward an integration. Psychoanalytic Dialog. 1, 121–147. doi: 10.1080/10481889109538889

[ref50] Nabizadeh A. Farhadi M. Rashid K. Kordnoghabi R. (2020). The effectiveness of tactical defenses neutralization in intensive short-term dynamic psychotherapy on defensive styles, anxiety, and fear of intimacy in non-clinical sample. J. Res. Psychol. Health 13, 24–39. doi: 10.22098/JRP.2020.1203

[ref51] Neborsky R. J. Ten J. (2018). Mastering intensive short-term dynamic psychotherapy. London: Routledge.

[ref52] Nolte T. Bolling D. Z. Hudac C. M. Fonagy P. Mayes L. Pelphrey K. A. (2013). Brain mechanisms underlying the impact of attachment-related stress on social cognition. Front. Hum. Neurosci. 7:816. doi: 10.3389/fnhum.2013.00816, PMID: 24348364 PMC3841757

[ref53] O’Rourke N. Hatcher L. (2013). A step-by-step approach to using SAS for factor analysis and structural equation modeling. Cary, North Carolina, USA: SAS Institute.

[ref54] Repic T. (2007). Fear of intimacy among married and divorced persons in association with physical abuse in childhood. J. Divorce Remarriage 46, 49–62. doi: 10.1300/j087v46n03_04

[ref55] Riggs D. S. (2014). Traumatized relationships: symptoms of posttraumatic stress disorder, fear of intimacy, and marital adjustment in dual trauma couples. Psychol. Trauma Theory Res. Pract. Policy 6, 201–206. doi: 10.1037/a0036405

[ref56] Rohner R. P. Filus A. Melendez-Rhodes T. Kuyumcu B. Machado F. Roszak J.. (2019). Psychological maladjustment mediates the relation between remembrances of parental rejection in childhood and adults’ fear of intimacy: a multicultural study. Cross-Cult. Res. 53, 508–542. doi: 10.1177/1069397118822992

[ref9001] Ryff C. D. (1989). Happiness is everything, or is it? Explorations on the meaning of psychological well-being. J. Pers. Soc. Psychol. 57, 1069–1081. doi: 10.1037/0022-3514.57.6.1069

[ref57] Safari Mousavi S. Sadeghi M. Sepahvandi M. A. (2021). The factor structure and psychometric properties of Mentalization questionnaire: a self-report measure for the assessment of Mentalizing capacity. Res. Cogn. Behav. Sci. 10, 123–134. doi: 10.22108/CBS.2021.127401.1492

[ref58] Schreiber J. B. Nora A. Stage F. K. Barlow E. A. King J. (2006). Reporting structural equation modeling and confirmatory factor analysis results: a review. J. Educ. Res. 99, 323–338. doi: 10.3200/joer.99.6.323-338

[ref59] Senese V. P. Miranda M. C. Lansford J. E. Bacchini D. Nasti C. Rohner R. P. (2020). Psychological maladjustment mediates the relation between recollections of parental rejection in childhood and adults’ fear of intimacy in Italy. J. Soc. Pers. Relat. 37, 1968–1990. doi: 10.1177/0265407520912339

[ref60] St Clair M. (2004). Object relations and self-psychology: An introduction. Belmont, Ca: Thomson/Brooks/Cole.

[ref61] Tabachnick B. G. Fidell L. S. Ullman J. B. (2013). Using multivariate statistics, Vol. 6. Boston, MA: Pearson, 497–516.

[ref62] Taubner S. Curth C. (2013). Mentalization mediates the relation between early traumatic experiences and aggressive behavior in adolescence. Psihologija 46, 177–192. doi: 10.2298/psi1302177t

[ref64] Vangelisti A. L. Beck G. (2007). “Intimacy and fear of intimacy,” in Low-cost approaches to promote physical and mental health. ed. L’Abate L. (New York, NY: Springer).

[ref63] Van Meter F. Handley E. D. Cicchetti D. (2020). The role of coping strategies in the pathway between child maltreatment and internalizing and externalizing behaviors. Child Abuse Negl. 101:104323. doi: 10.1016/j.chiabu.2019.104323, PMID: 31935532

[ref65] Venta A. Hatkevich C. Mellick W. Vanwoerden S. Sharp C. (2017). Social cognition mediates the relation between attachment schemas and posttraumatic stress disorder. Psychol. Trauma Theory Res. Pract. Policy 9, 88–95. doi: 10.1037/tra0000165, PMID: 27336218

[ref66] Vrtička P. Vuilleumier P. (2012). Neuroscience of human social interactions and adult attachment style. Front. Hum. Neurosci. 6:212. doi: 10.3389/fnhum.2012.00212, PMID: 22822396 PMC3398354

[ref67] Wais M. (2022). Mentalizing as a mediator between sexual abuse and PTSD in adolescents (Doctoral dissertation). Corpus ULaval. Canada: Université Laval. 73597. Available at: http://hdl.handle.net/20.500.11794/73597

[ref68] Williamson S. (2009). The relationship between severity of childhood sexual abuse and adult perceptions of intimacy with internalized shame as a mediator (master’s thesis). Brigham Young University - Provo, UT, BYU ScholarsArchive. 1875. Available at: https://scholarsarchive.byu.edu/etd/1875

[ref69] Wise J. E. (2016). Intimacy versus isolation. Intimacy Post-Injury, Canada: Combat Trauma and Sexual Health, Oxford University Press.

[ref70] Zhong J. (2023). On the theory of self-structure I: The nature of narcissism. Psychoanalysis and Psychotherapy in China 6, 15–36. doi: 10.31234/osf.io/753yp

